# Norwegian PhD students' discourses about practice‐relevant research in the field of health and social work

**DOI:** 10.1002/nop2.432

**Published:** 2019-12-26

**Authors:** Margareth Kristoffersen, Åse Vagli, Bjørg Frøysland Oftedal

**Affiliations:** ^1^ Department of Care and Ethics Faculty of Health Sciences University of Stavanger Stavanger Norway; ^2^ Department of Social Studies Faculty of Social Sciences University of Stavanger Stavanger Norway; ^3^ Department of Quality and Health Technology Faculty of Health Sciences University of Stavanger Stavanger Norway

**Keywords:** discourse analysis, health and social work professionals, knowledge discourse, PhD student, practice‐relevant research, professional knowledge

## Abstract

**Aim:**

The aim of this study was to explore the discourses of PhD students concerning the performance of practice‐relevant research in health and social work.

**Design:**

An explorative, qualitative design and a discourse analytical approach were used to collect data.

**Methods:**

Participants were recruited from a national research school for practice‐relevant research in Norway. Group interviews with ten PhD students (five health and five social work professionals) with extensive, wide‐ranging work experience as practitioners were used to collect data. The data were analysed by discourse analysis.

**Results:**

The analysis revealed three discourses: (a) the professional knowledge discourse; (b) the promoting partnership discourse; and (c) the using research knowledge discourse. The discourses are explicit concerning how systematically formed practice‐relevant research in health and social work is linked to dominant discourses about knowledge in the scientific field. Professional knowledge, promoting partnership and using research knowledge are fundamental features of practice‐relevant research.

## 1 INTRODUCTION

High‐quality doctoral education is a significant issue worldwide, especially in Western countries (Jablonski, 2001; Jones, 2018; Lee, Brennan, & Green, 2009; Maynard, Vaughn, Sarteschi, & Berglund, 2014; Scott, Brown, Lunt, & Thorne, 2004; Taylor, 2007), where the emergence of higher education studies primarily occurred after the Second World War (Macfarlane & Burg, 2019). The PhD students in this study were carriers of certain types of knowledge because they were professionals and academicians in health or social work. They had educational backgrounds and work experience as professional practitioners and were attending a recently initiated cross‐disciplinary research school. The research school was established as a collaboration among four universities in Norway, where the PhD programmes for professionals in practice‐oriented disciplines such as health and social work are promoted by higher education policies and the need for a well‐educated work force (St.meld.7 2014–2015; St.meld.18 2014–2015). The national research school is the only practice‐relevant research organization in Norway that aims to educate constructively critical and action‐directed researchers for complex and changing practice. This educational aim involves developing PhD students' abilities to identify, analyse and evaluate practice‐relevant research questions. Additional educational aims are to contribute to an increased understanding of the differences and similarities between professions, to develop knowledge‐based practice and to foster the ability to implement relevant scientific knowledge in health and social work.

Practice‐relevant research occurs in a multi‐professional context, transcends disciplinary boundaries and bridges science and practice (Frogett & Briggs, 2012; Hoffman, Pohl, & Hering, 2017). Such contexts can involve a plurality of theoretical approaches and contain discrepancies between theories and their application. The term “practice‐relevant” research therefore refers to issues of relevance for professions and practices (Fillery‐Travis & Robinson, 2018) and processes or activities that aim to influence, change and validate practice and it is relevant for professional practitioners and service users (Frogett & Briggs, 2012). Partners in the field are involved in all steps of the research process to guarantee practice relevance, and there is emphasis on implementing research results in practice (Gray, Sharlane, Heinsch, & Schubert, 2014; Heinsch, Gray, & Sharland, 2016). Practice‐relevant research aims to advance the body of scientific knowledge to meet practice needs.

Few empirical studies have investigated PhD students' discussions of the process of conducting practice‐relevant research. Thus, there is a need to study the discourses to which PhD students who are professionals and academicians relate and that they address in their discussions. More precisely, it is relevant to explore the discourses of PhD students to identify the dominant knowledge accounts involved in the process of conducting practice‐relevant research. A “discourse” can be described as a mindset or a set of common assumptions held by a group of people (Moses & Knutsen, 2012). Accordingly, a discourse is impossible without knowledge. In other words, discourses can be considered human meaning‐making processes that produce frameworks of meaning through the use of words, the practice of reflexivity and the creation of accounts (Wetherell, Taylor, & Yates, 2001). The accounts are seen as an expression of cultural standardized knowledge associated with particular social settings (Miller, 1997) and can be used to reveal *the positions and functions that the subject could occupy in the diversity of discourse within a particular field* (Foucault, 1972, 200). Therefore, the PhD students' articulations are perceived to be formed by the discursive vocabularies of scientific knowledge and debates, which constitute the context where the PhD students are framed. In the words of Miller and Silverman (1995), such an approach involves making institutional discourses available through discussion as the PhD students describe, negotiate and justify their preferred versions of social reality. However, this does not include the investigation of differences between discourses of knowledge in the health and social sciences (i.e. how dominant biomedical discourses differ from disciplinary discourse in nursing or social work) but rather an exploration of the discourses of PhD students involved in the process of conducting practice‐relevant research. The content of their discourses about knowledge have relevance for debates about research in health and social work.

## 2 BACKGROUND

There have been several attempts in the research literature to determine what characterizes discourses about knowledge (Brante, 2013, 2014). A dominant discourse describes practice as actual applications of knowledge and the scientific knowledge bases from which professionals derive their status (Brante, 2014; Moos & Krejsler, 2006). Both health and social work professionals are often described as lacking a specified scientific knowledge base (Brante, 2013; Etzioni, 1969; Fillery‐Travis & Robinson, 2018; Risjord, 2010). It has been argued that professions need a unique knowledge base as a signifying property that supports independent actions (Brante, 2011). For instance, Abbott (1988) notes that professionals have skills, usually abstract skills requiring extensive training, that can be applied case by case and not in a purely routine fashion. There are many disputes regarding this dominant discourse about knowledge.

One dispute notes the lack of integration between being science‐based as an academician and as a professional; rather, these points of departure are in opposition to each other (Brante, 2014). This position emphasizes the need to strengthen professions by establishing a scientific knowledge base and often focuses on the necessity of disciplinary boundaries, that is the epistemic grounds on which professionals anchor their claims of academic legitimacy and their defined, distinctive knowledge bases (Brante, 2010; McNamara, 2009; Risjord, 2010; Trevithick, 2008). Another dispute addresses the status of professionals in academia and the status of academicians in professional practice (Domakin, 2014; Kelly, 2017; McNamara, 2009; Risjord, 2010). This position has highlighted that a lack of professional knowledge can be problematic for researchers in academia when building relationships with professional practitioners. Accordingly, it can be problematic for health and social work professionals to relate to academia, as health and social work professions are regarded as multi‐paradigm, are more fragmented with heterogeneous scientific knowledge bases and are context‐oriented (Brante, 2010, 2014; McNamara, 2009). A third dispute concerns the theory‐practice gap debate, which is largely connected to two claims: practitioners make too little use of research and researchers pay too little attention to making research results known and useful (Freshwater, 2004; Gray et al., 2014; Risjord, 2010; Domakin, 2014). These claims can further be related to the knowledge use discourse (Freshwater, 2004; Gray et al., 2014; Taylor & Rafferty, 2003) addressing processes through which knowledge is produced and used in practice (Gray et al., 2014). This study aims to explore the discourses of PhD students concerning the performance of practice‐relevant research in health and social work.

## 3 MATERIALS AND METHODS

### 3.1 Participants

Non‐probability sampling was conducted by means of convenience sampling procedures (Polit & Beck, 2017). The inclusion criteria were PhD students attending the selected research school, which aims to support a 3 or 4 year regular PhD programme (a 4 year programme includes a service at the university, i.e., as a lecturer or supervisor for bachelor's or master's degree students). PhD students (*N* = 22) were invited by the administrative leader of the school to participate in the study.

Ten Norwegian PhD students with either health or social work professional backgrounds agreed to participate. The participants included nine women and one man. This gender split was representative of the PhD students at the research school. The participants were aged between 30–62 years (median 41 years) and had disciplinary knowledge and extensive, wide‐ranging professional work experience of approximately 6–40 years. The participants were in the first or middle stage of their PhD studies. Their PhD theses reflected various profession‐ and practice‐relevant research problems.

### 3.2 Data collection

The data were collected through group interviews (Fontana & Frey, 2005; Malterud, 2017) in 2016. The participants were divided into three groups, each group consisting of three or four participants. Each group met once for up to one or one and a half hours. The group discussions, which took place at the university during a research school gathering, were conducted in Norwegian, audiotaped and carefully transcribed verbatim.

Group discussions were chosen to come as close as possible to *everyday speech acts in conversations* (Kamberelis & Dimitriadis, 2005). Capturing the participants' responses in real space and time in the context of face‐to‐face interactions was particularly important, as this type of data comes closest to the idea of “naturally occurring” data (Peräkylä, 2005, 870). To assess the way the participants talked about the process of doing practice‐relevant research, one interviewer (MK) asked the initial question and the other interviewer (BFO) had a specific focus for the follow‐up questions. The initial question asked the group to discuss practice‐relevant research and the properties or features associated with the research process. To encourage the participants to discuss freely and at length about the research theme, the interviewers asked the participants to expand on what they had already said. By asking open questions, the interviewers largely avoided leading the participants' answers. More specific questions were also posed, such as the following: “What are the features of practice‐relevant research?” “How do you define practice‐relevant research or how do you argue for your research?” and “How has the research process been conducted since you started at the research school?” All participants actively engaged in the group discussions. They both supported and challenged each other, introducing opportunities for multiple meanings and interactions, which meant that the discussions served as consolidations of perspectives and interactions.

### 3.3 Ethical considerations

The study was approved by The Norwegian Centre for Research Data (no. 43829). Verbal and written information about the study was given by the administrator to all the PhD students at the research school. Those who wished to participate in the study were asked to send an e‐mail to one of the researchers and written consent was obtained before participation. The participants were assured confidentiality and notified about their right to withdraw.

### 3.4 Discourse analysis

The discourse analysis approach, which rests on an ethnomethodological discourse analytical tradition (Miller & Silverman, 1995), considers discourses as human meaning‐making processes and focuses on language (Wetherell et al., 2001), such as the words and expressions the participants used in their accounts to describe the process of conducting practice‐relevant research.

The discourse analysis was based on 60 pages of transcripts of recorded interview material and was concerned with the careful notation of the words used and the interactive processes between the participants and the researchers doing the interviews. To create a preliminary description, we listened to the discussions several times and read and re‐read the transcripts to closely examine what occurred during the discussions. Discourse analysis depends on an embodied attention and sensitivity related to talk (Silverman, 2006). Reflective questions were posed to identify which interpretative resources the participants used to describe their experiences (Miller, 1997). We looked for dominant patterns as well as for contrasts. Our reflective questions included the following: What did the participants talk about? What were the themes about doing practice‐relevant research and which subjects were emphasized? Which metaphors were used in the spontaneous articulation of meaning or, in other words, which figures of speech that were not literally true were used as descriptions of objects to explain what was being talked about? How did the participants position themselves in relation to doing practice‐relevant research? What was at stake? Which tensions were described? Where there any differences, agreements or disagreements between the health and social work professionals? Preliminary analysis involved extracts from each transcript being tentatively clustered by one of the researchers (MK). These were read again to condense and transform the data by identifying and describing the dominant discourses (Silverman, 2006). In the interpretation phase, relevant methodological literature was reviewed in relation to the descriptions, which were read and re‐read to reflect on parts of the text to interpret and identify the activated discourses, which are described as findings.

All three researchers (MK, ÅV and BFO) were involved in the analysis. It was important to appreciate possible interpretations while also identifying anything in the text that might contradict these interpretations. Several informal get‐togethers were arranged to ensure that we contributed to the analysis by reflecting on, describing and examining the descriptions of the text and identifying the discourses. This internal verification process was conducted continuously to maintain an open mind, be sensitive to nuances in the text and focus on the way the discourses constituted what the participants discussed (Silverman, 2006), indicating that the analysis was conducted appropriately and in such a way that the study's findings were validated.

### 3.5 Strengths and limitations

This study's trustworthiness is considered a strength, as the PhD students had knowledge of the research topic and everyday experiences of important issues involved in doing practice‐relevant research. Their PhD projects reflected different practice‐relevant research problems. Nonetheless, the study's relatively small‐scale and the lack of definitional and conceptual clarity in the field might be obstacles to the study's capacity to offer information about what practice‐relevant research involves (Heinsch et al., 2016).

The data collection method was suitable and the data were derived from discussions involving the participants' detail‐rich language about conducting profession‐ and practice‐relevant research. In addition, the discussions from which the data stemmed were created relationally (Dahlberg, Dahlberg, & Nyström, 2008) and in study‐life conditions. Multiple discourses were used, including arguments against each other to elaborate their discussions (Lincoln & Guba, 1985; Peräkylä, 2005). Features of practice‐relevant research were contained in the discourses. However, it is conceivable that other PhD students without work experiences as professionals might have discussed issues other than those mentioned by the participants.

The data may be considered limited for reasons that were impossible to resolve. Initially, the participants were divided into three groups with three and four participants in each group, but there were two participants for two interviews and six participants for the third interview. However, it is a strength that all the participants engaged in lively discussions with each other and had established relationships with each other prior to their discussions (Flynn, Albrecht, & Scott, 2018). The participants nonetheless may have been less open than they could have been because two researchers were present and represented a certain kind of expertise. These researchers were aware of their own roles in regard to follow‐up questions (Peräkylä, 2005; Silverman, 2006) and were not engaged in the PhD students' research work or at the research school. In addition, as highlighted by Silverman (2006), the researchers were aware that multiple versions of the world are legitimate and discussions are open to multiple readings.

## 4 FINDINGS

The analysis revealed three discourses: (a) the professional knowledge discourse; (b) the promoting partnership discourse; and (c) the using research knowledge discourse.

### 4.1 Professional knowledge discourse

The PhD students talked about professional knowledge as helpful when conducting practice‐relevant research. This knowledge provided a knowledge base for the research and was referred to as a distinctive or unique knowledge base that was previously achieved as a health or social work professional.

The PhD students agreed about the advantages of having knowledge as a health or social work professional: “We are nurses and thus, I have a strong sense of being a nurse and we bring with us who we are as a person in addition to our profession.” This quote shows how the personal pronouns “we” and “I” were used to articulate agreement, implying that the students positioned themselves in relation to each other, as illustrated by expressions such as “I'm thinking of what you just said”, or “we have already stated that….” By supporting each other's utterances, the PhD students established relationships with each other that evidenced their consideration of others' viewpoints as important. This mutual support implies that they positioned themselves as practice‐relevant researchers by pointing out what they had in common: they were all health and social work professionals with professional knowledge.

The quality of professional knowledge was described as embodied and this embodiment shaped the way the students perceived professional knowledge as integrated and personal:

I admit that I'm basically a nurse and have a deep sense of being a professional. My eyes have been opened in regard to how much knowledge is embedded within my profession and what I have learned was developed in the years before I started my doctoral degree.

Embodied professional knowledge was said to be acquired through work experience as a health or social work professional. The PhD students' discourse noted that practical experience was an advantage that contributed to informed practice‐relevant research. Embodied skills contribute to designing significant, useful research. The discourse emphasized that practical experience as a health or social work professional prevents the loss of relevant data and enables researchers to see important layers in the data that another researcher might miss due to a lack of embodied professional knowledge:

A minimum amount of embodied experience as professional practitioner, yes, that's a strength. Research benefits from such experience because it leads to an embodied familiarity with the field and it becomes possible to ask ‘wise’ research questions of practical interest, not only theoretical ones.

The quality of professional knowledge was further elaborated using metaphors. The PhD students' use of metaphors can be understood as expressions of how their professional knowledge constituted embodied skills. In particular, the health professionals talked about the relationship between the body and professional knowledge by using rich metaphors such as “within my navel.” As a metaphor, “within my navel” can be seen as an area of practice characterized by a familiar point of view that is connected to where the PhD students came from and what they had accomplished; outside that area, there was uncertainty related to what was happening in terms of conducting practice‐relevant research. However, the greater research context outside that area of practice did not seem to represent so much uncertainty that the students avoided taking on new positions as PhD students:

At the beginning of my doctoral education, I had a strong sense of being ‘within my own navel’, meaning my own field of nursing, without being aware of a greater context than that

Another metaphor used was “spinal cord.” The spinal cord connects the brain to the human body and is part of the central nervous system. The use of this metaphor can be understood as an expression of how professional knowledge was embodied as content for doing practice‐relevant research. Setting oneself free from unique professional knowledge distinguishing practice‐relevant research from other types of research was rather problematic for PhD students. Professional knowledge was influential in addressing and understanding challenges in practice‐relevant research. It was described as follows:

As a researcher, I bring ‘the nurse's knowledge’ with me in my ‘spinal cord’ when, for example, I work with the interview guide and I use the ethical guidelines for nurses. So, I have a lot of benefits from having nursing skills and knowledge when I'm doing my research.

The influence of professional knowledge was emphasized through a story that described a researcher as “a human being sitting on a cloud.” The metaphor included descriptions concepts such as a person sitting on miniscule water droplets or something impossible to touch or sit on and, consequently, falling right through. This metaphor was used by the PhD students as a contrast to express how professional practitioners perceived a researcher who is an academician as lacking professional knowledge and relevant pre‐understanding. The PhD students stated that when they worked as professional practitioners, researchers without relevant pre‐understanding as health or social professionals would not be considered in the same class as professional practitioners. Status as an academician would not render this researcher capable of asking relevant research questions of practical interest. Without substantive, circumscribed professional knowledge, it would be difficult to position oneself as a researcher who understands what goes on in practice and to gain community and affiliation with professional practitioners. Knowledge about the health or social work professions was seen as essential to prevent poorly executed or trivial research:

You need knowledge about the profession and about features of professional knowledge and how such knowledge has been developed over time and you need knowledge about the history of the professions and the process of professionalization.

### 4.2 Promoting partnership discourse

Promoting partnership with several partners involved taking into consideration partners' expertise in health or social work and engaging them as collaborators in the research process (e.g. in relation to the topic, interventions and analysis). However, it was highlighted that access to research should be concerned with processes extending beyond the level of the individual's accountability to interactions and communications on an organizational level between the university and health or social work organizations. Formal research policy was indicated as a necessary basis for establishing a formalized collaboration: “It should be a commitment, meaning a research profile for the university and this should not be depend on the single researcher him‐ or herself.” Access to the research field was not taken for granted and could be rather problematic. The PhD students therefore asserted that such access should not depend on the individual student's knowledge and skills.

This discourse also highlighted how building partnerships with service users and involving them in the research process could facilitate a research strategy called “*bottom‐up research.”* Such a strategy was said to require flexibility when conducting research, but it was seen as “very positive that the service users, patients or next‐of‐kin should be involved as co‐producers in a research project.” The discourse nonetheless addressed criticism of service users' status as co‐researchers and their ability to participate in the research process. The PhD students questioned the involvement of service users as co‐researchers. They argued that taking accountability of such involvement was challenging because the users' knowledge and skills were possibly irrelevant and that training as a co‐researcher should be required to contribute to the research process.

Moreover, investing resources and engagement in collegial relations during practice‐relevant research was “a must.” The PhD students talked about collaboration as forming mutually beneficial relationships, which in turn could raise general awareness of research projects and render their results usable. Interacting, communicating and exchanging information with professional practitioners and having research skills were seen as key, particularly because the research context is complex and should be considered comprehensively:

It is important to study the whole context within the research field with all the problems and pleasures related to collaboration with professional practitioners. The context is important.

At the same time, the PhD students thought that involvement of professional practitioners required a balance between proximity and distance. The PhD students sometimes experienced a sense of being “a troublesome journalist” and having to ask practitioners many questions to collect data about a practice‐relevant research problem. Moreover, they thought that promoting partnership might require giving something in return to health and social work. As professionals, the PhD students were used to practising “an unwritten rule that one has to give something.” When organizational frames for research were not optimal, demonstrating gratefulness was considered necessary, even though it could be quite difficult to know what to give in return. One solution was to compensate for the time used by providing help with nursing care to reduce the professional practitioners' workloads:

Doing practice‐relevant research is difficult because the patients must be taken care of and then being a participant in a research project is one more demand for professional practitioners. So, I had to work as a nurse to care for the patients – it was kind of helping each other as colleagues. I know this wasn't how research should be done, but this was what I did to prevent the participants from having to use their leisure time to participate in the research project.

This quote explicitly attests to how practice‐relevant research refers to complex contextual processes. Promoting partnership with professional practitioners did not necessarily correspond to the requirements of other types of research. Researchers who were not capable of performing any professional skills that required extensive training and that were described as “a craft” or “work of a craftsman” (3) were at risk of being excluded. Practice‐relevant research was considered inaccessible and not suitable for researchers lacking the capability to give something in return to professional practitioners. More concretely, a “paper and pen”‐based research strategy was not deemed adequate for the execution of such research:

Doing ‘paper and pen’ research only is useless. It is impossible sitting in an office with books and theoretical perspectives; instead, you need to know where to focus and which questions to ask and you have to collect the data. So, you can't just sit down and wait for the answers.

The PhD students perceived professional skills to be essential and saw behavioural and attitudinal flexibility as necessary for their confidence as researchers in health or social work. This required the ability to use analytical judgement when planning and conducting well‐designed practice‐relevant research projects in accordance with relevant research traditions and mindfulness of many things at once and thus could be quite exhausting.

### 4.3 Using research knowledge discourse

The PhD students assumed accountability for the application of research results. It was noted that through innovation, practice improves, which is the overall purpose of practice‐relevant research. The PhD students indicated that achieving impacts in health or social work was enjoyable, suggesting that improving the quality of practice also develops the quality of the profession and thereby draws attention to the potential of the profession.

Nevertheless, the discourse emphasized practice as the domain of the professional practitioner and positioned professional practitioners as capable of using research results and taking responsibility for improvements. Professional practitioners were viewed as engaged in their work, able to maintain it and having the knowledge and embodied skills to perform it, thus indicating that they should be respected:

The competence of the practitioner develops over many years; he or she is a master and it is important to continue doing what they manage to do. So, as a researcher you have to understand this point.

The implementation of research results required the use of advanced professional skills to identify and analyse the challenges involved in innovation processes. The PhD students' discourse demonstrated resistance to being accountable for introducing and implementing changes in practice. They did not want to be “change agents” and positioned professional practitioners as capable of making changes. Forcing invalid results onto health and social work was considered useless. The PhD students believed that such practices do not necessarily provide fruitful collaboration with professional practitioners and often result in implementations unrelated real problems in the field.

## 5 DISCUSSION

This discourse analysis of three PhD student group discussions has revealed that conducting practice‐relevant research in health and social work is associated with specific features: professional knowledge, promoting partnership and using research knowledge.

The discourses of PhD students highlighted professional knowledge as one knowledge base that distinguishes practice‐related research from other kinds of research. It was indicated that professional knowledge was influential in addressing and understanding challenges in practice‐relevant research in health and social work. Health and social work professionals' knowledge and skills provide a distinctive epistemic ground that are considered relevant in legitimatizing practice‐relevant research. In fact, the PhD students asserted that it is essential for research. Their accounts contribute to the dominant discourse about knowledge that suggests the importance of having a specific disciplinary knowledge base (Brante, 2014, 2011; Heinsch et al., 2016; McNamara, 2009; Moos & Krejsler, 2006; Risjord, 2010). Without undermining the dispute about the lack of integration between being science‐based as an academician and as a professional (Brante, 2014; 2011), professional knowledge was, to a great extent, discussed as being aligned with and not in opposition to research skills. Practice involves the application of knowledge and professional knowledge was said to be an advantage. This type of specialization can, in the view of Friedson (2001), be understood as both essential to practice‐relevant research and as complementary to academia and practice in its performance. Professional knowledge seemed to anchor the PhD students' claims of academic legitimacy and promote practice relevance. A carrier of professional knowledge understands what goes on in practice and can achieve community with professional practitioners. Professional knowledge contributes to reducing conflict between academia and practice and, more broadly, to the two fields being viewed as complementary to each other. However, the embodiment of professional knowledge is seen as inherent. The PhD students' discussions indicated that practical experience as a health or social work professional contributed to the embodiment of professional knowledge. Embodied ways of knowing, provide adequate “embodied know‐how”, that is, experiential or tacit knowledge not easily articulated (Benner, 2000) in the process of doing practice‐relevant research. Thus, the integrated and personal quality of professional knowledge seemed to enable the PhD students to be practice‐relevant researchers and assisted them in conducting research.

Furthermore, the PhD students emphasized that conducting practice‐relevant research requires a qualified researcher with relevant pre‐understanding to collaborate with partners, that is professional practitioners and service users in all steps of the research process. The promoting partnership discourse supported the dispute about the status of professionals in academia and the status of academicians in professional practice (Domakin, 2014; Kelly, 2017; McNamara, 2009; Risjord, 2010). The discourse recognized researchers' status based on the research skills that met needs in health or social work and produced a cultural context of domination. Specifically, professional knowledge and skills had status, while being an underqualified researcher (i.e. not having professional skills that promote collaboration with partners in all steps of the research process) was not seen as allowing one to be a practice‐relevant researcher. As noted by Hockey (1994), Murakami‐Ramalho, Militello, and Piert (2013), becoming a researcher seems to rely partly on the knowledge and skills one already has. In Friedson's (2001) and Abbott's (1988) view, a circumscribed body of knowledge is an absolute advantage in promoting partnership to conduct practice‐relevant research. It might involve skills that usually require extensive training to be applied case by case and not in a purely routine manner. A qualified researcher is required, for example, when organizational frames for research are not optimal and the PhD candidates must provide something in return to compensate for the professional practitioners' time spent participating in research. Thus, practice‐relevant research in health and social work involves complex contextual processes and does not necessarily correspond to other kinds of research. As professionals derive status from scientific knowledge bases (Brante, 2014, 2011; Domakin, 2014; Kelly, 2017; McNamara, 2009; Moos & Krejsler, 2006; Risjord, 2010), it is considered necessary to be an insider in research (Costley & Pizzolato, 2018). Accordingly, not being educated as a health or social work professional when doing practice‐relevant research was considered incompatible with taking accountability. Any research undertaken by such individuals was considered likely to be doomed and invalid. Lack of previous professional experience could have several negative effects related to the research process, for example, the recruitment of participants, involvement of partners and implementation of research results.

Nonetheless, in the using research knowledge discourse, the researcher's status as a change agent was questioned, implying that the results contribute to the debate on the theory‐practice gap debate (Freshwater, 2004; Gray et al., 2014; Risjord, 2010; Domakin, 2014). The PhD students' accounts supported the claim that researchers might pay little attention to making research results known and useful. They subordinated the practice‐relevant researcher's status in relation to that of professional practitioners, and thereby, they also subordinated their own status as a PhD student related to be a change agent. Making research results known and useful was not articulated as the practice‐relevant researcher's task; instead, the ability to consider the usability of research results was asserted as being the responsibility of professional practitioners. The PhD students' discourse involved the practitioners' ability to implement research results related to real problems in health or social work. However, the PhD students had some willingness to accept responsibility for the use of research knowledge, indicating that they paid attention to the issue of how to be a change agent.

### 5.1 Implications

This study adds conceptual understanding to the work of practice‐relevant research. Although cross‐disciplinary qualities have relevance (Costley & Pizzolato, 2018), distinctive professional knowledge bases seem to be needed to conduct practice‐relevant research. Thus, careful attention should be paid to integrating such aspects in the term “practice‐relevant” research in discourse about knowledge in the health and social sciences.. Moreover, the term “practice‐relevant research” must be presented in a form that is relevant and renders it usable (Klenowski, Ehrich, Kapitzke, & Trigger, 2011; Trevithick, 2008). This means incorporating what is expected of high‐quality research (Ampaw & Jaeger, 2011; Lee & Murray, 2015) concerning how to perform as a researcher to practice research skills (Baker & Lattuca, 2010; Maynard et al., 2014) for betterment in changing practices (Marginson, 2016). One specific responsibility might be to appreciate the impact of the embodiment of professional knowledge and promote partnership with partners in practice and use of research knowledge.

## 6 CONCLUSION

This study makes explicit how systematically formed practice‐relevant research in health and social work is seen as linked to discourses about knowledge in the scientific field. As emphasized by the PhD students, professional knowledge, promoting partnership and using research knowledge seem to be fundamental features of practice‐relevant research. Making this finding visible is important, particularly because practice‐relevant research involves multifaceted processes in the highly complex field of health and social work. However, research on discourses about practice‐relevant research and distinctive knowledge bases for such research is in its beginning stages and needs to be elaborated in the future.

## CONFLICT OF INTEREST

There is no conflict of interest.

## AUTHOR CONTRIBUTIONS

Margareth Kristoffersen (MK) and Bjørg Frøysland Oftedal (BFO): Study design and data collection. MK: Data analysis and drafting the manuscript. Åse Vagli (ÅS) and BFO: Validation analysis. All authors contributed to editing the final manuscript, revised it critically for scientific content, read and approved the final version.
